# Alleviating Effects of Vitamins C and E Supplementation on Oxidative Stress, Hematobiochemical, and Histopathological Alterations Caused by Copper Toxicity in Broiler Chickens

**DOI:** 10.3390/ani11061739

**Published:** 2021-06-10

**Authors:** Mohamed A. Hashem, Sahar S. Abd El Hamied, Eman M. A. Ahmed, Shimaa A. Amer, Aziza M. Hassan

**Affiliations:** 1 Clinical Pathology Department, Faculty of Veterinary Medicine, Zagazig University, Zagazig 44511, Egypt; mhashem.vet@gmail.com; 2 Animal Health Institute, Zagazig Branch, Zagazig 44511, Egypt; sahar_elmasry91@yahoo.com (S.S.A.E.H.); emanalilogy@gmail.com (E.M.A.A.); 3 Department of Nutrition & Clinical Nutrition, Faculty of Veterinary Medicine, Zagazig University, Zagazig 44511, Egypt; 4 Department of Biotechnology, College of Science, Taif University, P.O. Box 11099, Taif 21944, Saudi Arabia; a.hasn@tu.edu.sa

**Keywords:** copper toxicity, vitamin C, vitamin E, hematological parameters, oxidative stress, histopathology

## Abstract

**Simple Summary:**

Excessive copper in diets is associated with numerous disadvantageous impacts on poultry. The current study evaluated the efficacy of vitamin C and vitamin E in mitigating oxidative stress, hematobiochemical, and histopathological changes in the kidney induced by copper sulfate (CuSO_4_) toxicity in broiler chickens. The birds were assigned to five experimental groups: 1st group—basal diet with no additives (control group), 2nd group—basal diet complemented with CuSO_4_ (300 mg/kg diet), 3rd group—basal diet with CuSO_4_ (300 mg/kg diet) + vitamin C (250 mg/kg diet), 4th group—basal diet with CuSO_4_ (300 mg/kg diet) + vitamin E (250 mg/kg diet), and 5th group—basal diet with CuSO_4_ (300 mg/kg diet) + vitamin C (250 mg/kg diet) + vitamin E (250 mg/kg diet). The current study’s findings showed the possible preventive impacts of dietary antioxidants on hematobiochemical alterations, oxidative stress, and kidney damage induced by CuSO_4_ toxicity.

**Abstract:**

The current investigation evaluated the alleviating effects of vitamin C and vitamin E on oxidative stress, hematobiochemical, and histopathological changes in the kidney induced by copper sulfate (CuSO_4_) toxicity in chickens. Two hundred and fifty-one-day-old male broiler chicks were randomly allotted into five experimental groups (five replicates/group, ten chicks/replicate): 1st group—basal diet with no additives (control group), 2nd group—basal diet complemented with CuSO_4_ (300 mg/kg diet), 3rd group—basal diet with CuSO_4_ (300 mg/kg diet) + vitamin C (250 mg/kg diet), 4th group—basal diet with CuSO_4_ (300 mg/kg diet) + vitamin E (250 mg/kg diet), and 5th group—basal diet with CuSO_4_ (300 mg/kg diet) + vitamin C (250 mg/kg diet) + vitamin E (250 mg/kg diet) for a 42 day feeding period. The results showed a significant reduction in red blood cells (RBCs), hemoglobin (Hb) concentration, and hematocrit values as well as total leukocyte counts (WBCs), lymphocyte, heterophil, and monocyte counts in the CuSO_4_-intoxicated birds (2.42 × 10^6^/µL, 9.54 g/dL, 26.02%, 15.80 × 10^3^/µL, 7.86 × 10^3^/µL, 5.26 × 10^3^/µL, and 1.18 × 10^3^/µL, respectively, at the 6th week) compared to (2.79 × 10^6^/µL, 10.98 g/dL, 28.46%, 21.07 × 10^3^/µL, 10.84 × 10^3^/µL, 7.12 × 10^3^/µL, and 1.60 × 10^3^/µL, respectively) in the control group. Moreover, CuSO_4_-intoxicated birds showed hypoglycemia with a rise in serum uric acid and creatinine levels (122.68, 5.18, and 0.78 mg/dL at the 6th week) compared to (159.46, 4.41, and 0.61 mg/dL) in the control group. The CuSO_4_ toxicity in birds induced oxidative stress, indicated by a high serum malondialdehyde level (MDA) and diminished activity of the antioxidant enzymes (glutathione peroxidase (GSH-Px) and superoxide dismutase (SOD)) (2.01 nmol/mL, 37.66 U/mL, and 2.91 U/mL, respectively, at the 6th week) compared to (1.34 nmol/mL, 57.00 U/mL, 4.99 U/mL, respectively) in the control group. High doses of Cu exposure caused severe microscopic alterations in kidney architecture. The addition of vitamins C and E, singularly or in combination, displayed a beneficial effect in alleviating these harmful effects of Cu toxicity. These findings showed the possible mitigating impacts of dietary antioxidants on the hematobiochemical alterations, oxidative stress, and kidney damage induced by CuSO_4_ toxicity.

## 1. Introduction 

Copper is a vital micromineral in living animals’ diets. It is essential for cellular metabolism and enzyme activity like Cu–Zn superoxide dismutase, tyrosinase, lysyl oxidase, and cytochrome C oxidase [1,2], which are engaged in a range of vital processes necessary for growth and maturation [2]. Supplementing diets with organic and inorganic Cu sources has beneficial effects on poultry production [3,4,5,6]. Attia et al. [7] reported that supplementing inorganic Cu (8 mg/kg) is better for growth of male White Pekin ducks from 1–56 d of age than inorganic Cu. Moreover, Cu supplementation increased plasma Cu and cholesterol and decreased Zn levels. They also reported improved liver Cu concentration and Cu excretion and retention by organic Cu supplementation compared to inorganic form. Copper supplementation (10 mg/kg) was sufficient for productive and reproductive performance and egg quality of laying hens [6]. Copper sulfate is the most common form used as a feed additive in poultry and livestock feed [8]. Supplementation with up to 250 ppm Cu has been proven for growth stimulation [1]. However, supplementation in excess amounts lowers growth, feed intake, and the feed conversion ratio in broiler chickens [9,10]. Several studies have demonstrated the toxic impacts of copper on broiler chickens [9,10,11]. The addition of 325 ppm copper to poultry diets initiates growth retardation and muscle atrophy [12]. The increased intake of copper in the feed results in morphological alterations in the visceral organs [6,13,14]. In our previous study, we reported alterations in the liver tissues in Cu-intoxicated chickens represented by hyperplastic and necrotic biliary epithelium with various degenerative and necrotic changes at the third week. Also, cholestasis, necrotic bile duct epithelia, in addition to lymphocytic portal aggregation and fibroblast proliferation, were encountered at the 6th week [15]. Chronic exposure also leads to hemolytic anemia and affects the central nervous system [14,16]. Exposure to excessive levels of Cu can lead to oxidative stress in broiler chickens [17,18], decrease the activities of copper–zinc superoxide dismutase (SOD), glutathione peroxidase (GSH-Px), and enhance the malondialdehyde (MDA) contents in ducklings [19]. 

When copper is found in supply more than cell’s demands, it stimulates the creation of free radicals and the immediate oxidation of fats, proteins, and DNA [20]. Numerous tools have been suggested to justify copper-induced cytotoxicity [2]. The base for these concepts is that free copper ions behave as potent catalysts for creating reactive oxygen species (ROS) [2,21]. Cupric and cuprous copper ions can perform an essential part in redox reactions. Such as, cupric ions (Cu^2+^) can be reduced to cuprous (Cu(^+^ in the existence of superoxide (O2^-^), which can catalyze the production of reactive hydroxyl radicals (OH) from hydrogen peroxide (H_2_O_2_) breakdown through the Haber–Weiss reaction [22]. Hydroxyl radicals are the most potent oxidative radicals potentially present in living systems and may stimulate lipid peroxidation causing tissue injury [23]. These outcomes can be diminished by antioxidant protection systems such as catalase, glutathione peroxidase, superoxide dismutase, and vitamins C and E [21,24].

The dietary addition of various antioxidants such as vitamin C, vitamin E, and selenium was efficiently practiced, mitigating oxidative stress in vivo and in animal products [25,26,27,28,29]. Vitamin E is a coating antioxidant that protects the intracellular structures of live organisms. It acts through mitigating the toxic effects of free radicals and reactive oxygen species that motivate oxidation of phospholipids and sulphydryl groups, leading to an impaired cell membrane structure [30]. Likewise, vitamin C is the most essential water-soluble antioxidant, as it protects biofilms from lipid peroxidation by removing peroxyl radicals in the aqueous stage before the oxidation process starts [30]. It also works to replenish the reduced vitamin E. Vitamin C cannot directly eliminate the lipophilic radicals formed in the membranes, but it reduces the number of tocopheroxyl radicals that adhere to the membrane during the transition of the lipophilic to the aqueous phase [31]. Numerous investigations disclosed good performance with supplementing of vitamins (C and E) in broiler chick diets [12,26,27,32] or fish [25,28,33,34]. Recently, different reports have been reported that vitamins E and C, due to the fact of their role as antioxidants, can protect against toxic infections from xenobiotics and those from minerals, too [35]. Both vitamins can function synergistically to inhibit the adverse outcomes of copper toxicity [12]. In our previous study, it was reported that a combination of vitamin C and E can mitigate the histopathological and DNA changes in the liver of CuSO_4_-intoxicated birds [15]. The present study aimed to evaluate the efficacy of single and combined supplementation of vitamin C and vitamin E in mitigating oxidative stress, erythrogram, leukogram changes, and histopathological alterations in the kidney induced by copper sulfate (CuSO_4_) toxicity in broiler chickens. 

## 2. Material and Methods 

### 2.1. Experimental Birds, Design

This study was conducted in a poultry research unit in the faculty of veterinary medicine, Zagazig University, Egypt. The ethics of the experimental protocol were approved by the Institutional Animal Care and Use Committee of Zagazig University, Egypt (ZU-IACUC/2020). All animal experiments were performed following the recommendations described in “The Guide for the Care and Use of Laboratory Animals in Scientific Investigations”.

Two hundred and fifty-one-day-old male broiler chickens (COBB-500) were attained from Al-Kahira Poultry Company, 10th of Ramadan City, Sharkia Governorate, Egypt. The experiment lasted for 42 days with good ventilation. Birds were bred in an open, well-ventilated house with sawdust. The chicks were stocked in pens with 10 birds each. A pen is considered a replicate. The room temperature was thermostatically controlled and regulated by two heaters. The room temperature during the first week was set at 34 °C and gradually decreased by 3 °C every week until it reached 24 °C. The lighting program for the first week was 24 h a day and then changed to 16 h of light and 8 h of darkness over 7–42 days. Freshwater and feed were accessible for ad libitum consumption throughout the experiment. The chicks were given a starter diet from day one until the 10th day of age, a grower diet (11th–22nd day), followed by a finisher diet until 42 days of age. The ingredients and chemical composition of the diets were formulated as defined in the COBB-500 Broiler Handbook [36] (Table 1). All birds were vaccinated at 7 and 14 days old against Newcastle disease and at 11 and 22 days old for Gumboro disease [37]. Birds were monitored for any disease challenge or mortalities.

The chicks were randomly allotted into five experimental groups (five replicates/group, ten chicks/replicate): the 1st group: basal diet with no additives (control group), the 2nd group: basal diet supplemented with CuSO_4_ (300 mg/kg diet), the 3rd group: basal diet with CuSO_4_ (300 mg/kg diet) + vitamin C (250 mg/kg diet), the 4th group: basal diet with CuSO_4_ (300 mg/kg diet) + vitamin E (250 mg/kg diet), and the 5th group: basal diet with CuSO_4_ (300 mg/kg diet) + vitamin C (250 mg/kg diet) + vitamin E (250 mg/kg diet) for a six-week feeding period. Copper sulfate (CuSO_4_·5H_2_O, El-Gomhoria Industry, Zagazig, Egypt), vitamin C (L-Ascorbic acid, phosphate, ROVIMIX^®^ STAY-C^®^35, DSM, Heerlen, Holland), and vitamin E (DL-α-tocopherol acetate, Pharco Pharmaceutical Industries, Zagazig, Egypt). The experimental diets were stored in a cool and well-ventilated place until used in the experiment. The toxic dose of Cu used in this investigation was determined according to Cinar et al. [12]. In contrast, the vitamins C and E doses were used as described by Sahin et al. [38].

### 2.2. Determination of Hematological and Biochemical Parameters

On the termination of the 3rd and 6th week, blood samples (two aliquots) were attained from the wing vein of (two birds/replicate, 10 birds/group). The first aliquot of blood was placed in tubes containing dipotassium salt of EDTA as an anticoagulant for hematological analysis by Hemascreen 18 Automatic Cell Counter (Hospitex Diagnostics, Sesto Fiorentino, Italy), including red blood cells (RBCs), hemoglobin (Hb), hematocrit (HCT), mean corpuscular volume (MCV), mean corpuscular hemoglobin (MCH), mean corpuscular hemoglobin concentration (MCHC), total leukocyte counts (WBCs), and differential leukocyte counts [18].

The second aliquot of blood was collected without anticoagulant, left to clot at room temperature, and centrifuged for 15 min at 1500 rpm for serum separation, which was stored at −20 °C in deep freezing until biochemical analysis. An automatic biochemical analyzer (Robotnik Prietest ECO-India) was used to measure the level of glucose, creatinine, and uric acid in the blood by following the described methods [39,40,41]. An UV-Vis Spectrophotometer (OPTIMA, PHOTOMECH. 301-D+, Japan) was used to estimate oxidative stress and antioxidants markers, including serum malondialdehyde (MDA), glutathione peroxidase (GSH-Px), and superoxide dismutase (SOD) according to the methods described in [42,43,44], respectively.

### 2.3. Histopathological Investigations

Kidney samples were harvested and fixed in 10% neutral formalin, fixative, dehydrated, and embedded in paraffin. Five-micron-thick paraffin slices were stained with hematoxylin and eosin (H&E) [45] and inspected microscopically. 

### 2.4. Statistical Analysis

Data were analyzed with one-way analysis of variance (ANOVA) using the GLM procedure in SPSS (SPSS Inc., Chicago, IL, USA), after the Shapiro–Wilk’s test was used to verify the normality and Levene’s test was used to verify the homogeneity of the variance components among experimental treatments. Tukey’s test was used to compare the differences between the means at a 5% probability. Variations in the data were expressed as the mean ± SD, and the significance level was set at *p* < 0.05.

## 3. Results 

### 3.1. Hematological Parameters 

As shown in Table 2, a significant decrease in RBCs, Hb concentration, and hematocrit (HCT) values were detected in the CuSO_4_-intoxicated birds compared with the CON group at the 3rd and 6th weeks (*p* < 0.05). Meanwhile, the MCV, MCH, and MCHC values displayed a non-significant change at the 3rd week indicating normocytic normochromic anemia, but at the 6th week only MCHC significantly decreased leading to normocytic hypochromic anemia. Compared with the CuSO_4_-intoxicated birds, dietary co-supplementation with vitamin C and E, singularly or in combination, led to amelioration in all changes in the erythrogram that returned towards the normal values of control all over the experimental period, especially in the CuSO_4_ + vitamin C + vitamin E group.

The WBCs, lymphocyte, and monocyte counts were significantly diminished in the CuSO_4_-intoxicated birds relative to the control group (*p* < 0.05). However, heterophil, eosinophil, and basophil counts were displayed non-statistical variations at the end of the 3rd week (*p* < 0.05). The same picture in the leukogram was observed at the termination of the 6th week, but the heterophil count significantly dropped (*p* < 0.05). Compared with the Cu-intoxicated group, supplementation of vitamin C and vitamin E to CuSO_4_-intoxicated birds improved the leukocyte picture in all groups, especially the CuSO_4_ + vitamin C + vitamin E group in which the WBCs, lymphocyte, heterophil, and monocyte counts were significantly increased and returned to the values of the control group (Table 3). 

### 3.2. Serum Biochemical Parameters

A significant decrease in serum glucose was found in Cu-intoxicated birds at the 3rd and 6th weeks. The uric acid and creatinine levels were not significantly different in all experimental groups at the 3rd week (*p* > 0.05). At the sixth week, the uric acid and creatinine levels in the copper intoxicated birds increased compared to the control group (*p* < 0.05). Vitamins-treated groups displayed favorable effects in serum glucose, creatinine, and uric acid levels that reverted close to the control values at the termination of the 6th week (Table 4).

### 3.3. Oxidative Stress and Antioxidant Status 

As evidenced in Table 5, CuSO_4_ toxicity in birds induced oxidative stress as indicated statistically by increased serum MDA levels and diminished SOD activity at the end of the 3rd and 6th week and decreased activity of GSH-Px at the 6th week compared with the control group (*p* < 0.05). However, the administration of vitamins induced a beneficial effect in the picture of the oxidative stress by significantly decreasing serum MDA levels and increasing the activity of GSH-Px and SOD compared with the Cu-intoxicated group.

### 3.4. Histopathological Findings 

**Figure 1:**
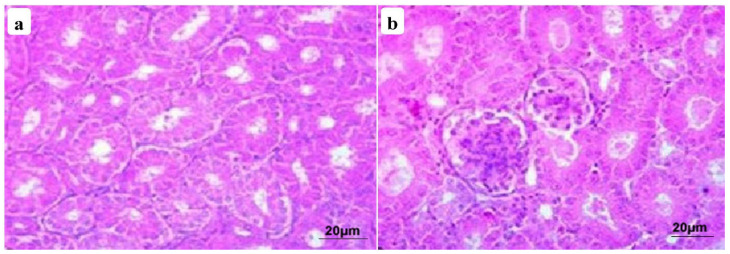
Photomicrographs of kidney sections from the control group at the 3rd week (**a**) and 6th week (**b**) showed normal histological structures.

**Figure 2:**
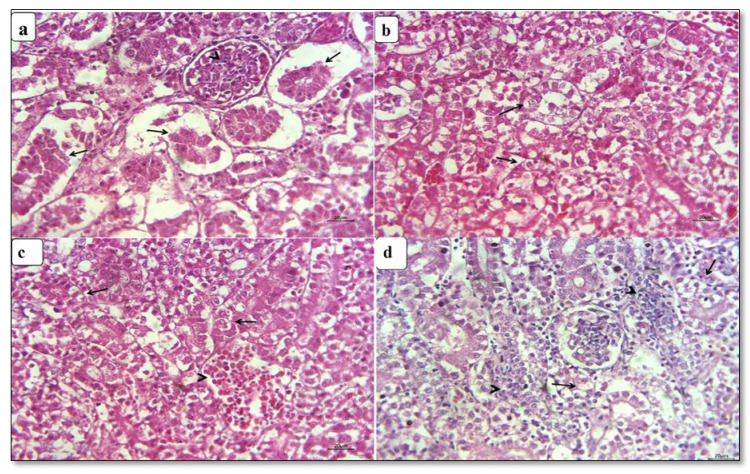
Photomicrographs of kidney sections from CuSO_4_-intoxicated and vitamin-treated groups at the 3rd week: (**a**) the kidney of CuSO_4_-intoxicated chickens shows extensive necrotic tubular epithelia (arrow) and hypercellularity of glomeruli (arrowhead); (**b**) the kidney of CuSO_4_ + vitamin C group shows moderate nephritic changes in renal tubular epithelia (arrow); (**c**) the kidney of the CuSO_4_ + vitamin E group shows various degenerative changes in some tubular epithelia (arrow) and partial intravascular hemolysis (arrowhead); (**d**) the kidney of CuSO_4_ + vitamin C + vitamin E group shows regenerative attempts from tubular epithelia (arrowhead) adjacent to nephritic tubules (arrow).

**Figure 3:**
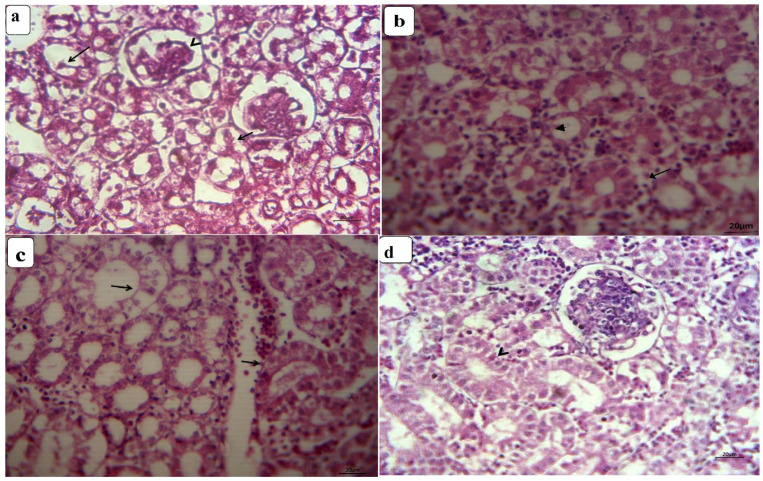
Photomicrographs of kidney sections from CuSO_4_-intoxicated and vitamin-treated groups at the 6th week: (**a**) the kidney of CuSO_4_-intoxicated chickens show pronounced oncotic necrosis of renal tubules (arrow) and glomeruli (arrowhead); (**b**) the kidney of the CuSO_4_ + vitamin C group show degeneration and necrosis of some tubular epithelia (arrow); (**c**) the kidney of the CuSO_4_ + vitamin E group shows normal renal parenchyma (arrowhead) with mild interstitial lymphocytic aggregation (arrow); (**d**) the kidney of the CuSO_4_ + vitamin C + vitamin E group shows normal nephron segments and intense regenerative attempts in the tubules (arrow).

The kidney of control chickens exhibited normal histological structures at the 3rd and 6th week (Figure 1a,b). In contrast, the kidney of CuSO_4_-intoxicated birds revealed nephrotoxicity represented by extensive necrosis of the renal tubular epithelial and hypercellularity of glomeruli at the 3rd week (Figure 2a). Moreover, extensive hemorrhage focally replaced renal parenchyma with congested blood vessels, and capillaries were detected, besides lymphocytic infiltrations in some glomeruli and hyaline cast inside tubular lumina. At the termination of the 6th week, renal damage became more extensive and characterized by pronounced oncotic necrosis of the renal tubules and glomeruli (Figure 3a), in addition to extensive hemorrhage and hemolysis were seen. Kidney of CuSO_4_ + vitamin C showed moderate nephrotic changes involving most renal tubular epithelia with few extravasated erythrocytes at the termination of 3rd week. In addition, most glomeruli restored their normal picture, and a few had necrosed glomerular tuft (Figure 2b). At the 6th week post-supplementation, moderate renal lesion represented by degeneration or necrosis of some tubular epithelia with hypercellularity of the glomeruli were encountered, with dilatation and hyperemia in the blood vessels (Figure 3b). Kidney of CuSO_4_ + vitamin E displayed various mild degenerative changes, mainly cloudy swelling or hydropic degeneration of tubular epithelia with partial intravascular hemolysis at the termination of 3rd week (Figure 2c). A few regenerative attempts in the tubular epithelia were detected. At the end of the 6th week, mild interstitial lymphocytic aggregation could be seen, with improvements of lesions in most renal parenchyma and the regeneration of histomorphology of all nephron segments (Figure 3c). Kidney of chickens supplemented with CuSO_4_ and both vitamins showed a reduction in renal toxicity. A few tubules still suffered from nephritic changes, while great regenerative attempts were encountered in the adjacent tubular epithelia at the end of 3rd week (Figure 2d). After six weeks of the experiment, the renal parenchyma had an intense reduction in lesions of copper toxicity. All segments of nephrons restored their normal histomorphologic picture with extreme regenerative attempts in the tubules and interstitium (Figure 3d).

## 4. Discussion

Copper is a vital element for animals, but dietary inclusion at high doses or over a long period can harm performance and increased lipid peroxidation [46]. Along with this experiment, no clinical signs were observed in all groups of birds, except the CuSO_4_–intoxicated group, which exhibited mild diarrhea, anorexia, and weight reduction with no mortalities. These results coordinated with other studies [47,48]. 

The erythrogram results in this study showed a significant reduction in RBC count, Hb concentration, and hematocrit value, whereas there were no significant changes in MCV, MCH, and MCHC values in copper-intoxicated birds at the 3rd and 6th weeks. The MCHC value significantly decreased only at the 6th week, indicating normocytic hypochromic anemia. Similar findings were reported in laying hens [46], turkeys [49], and Wister albino rats [50]. This anemia may be due to the excess copper bound to mucosal ferritin, interfering with the re-utilization of iron from ferritin in reticuloendothelial cells; thus, copper acted as a competitive inhibitor of iron, leading to iron-deficient anemia [51]. The decrease in Hb concentration could be due to the interaction of copper with copper-containing enzyme cytochrome oxidase, which is involved in heme synthesis by reducing Fe^+3^ to Fe^+2^ [52]. A recent investigation stated that intravascular hemolysis is a typical sign of copper toxicity resulting in a reduced Hb level [53]. Supplementation with vitamin C and vitamin E showed an improvement in the hematological values. The enhancing action of vitamins may be due to the fact of their roles as antioxidants, protecting the cell membranes of red blood cells against oxidative damages induced by heavy metal toxicity in chicks [54,55,56]. 

Regarding the results of the leukogram, the CuSO_4_-intoxicated birds showed leukopenia, lymphopenia, heteropenia, and monocytopenia. These results may be attributed to general injury produced by the toxic dose of CuSO_4_ on the hematopoietic stem cells in the bone marrow and other erythropoietic organs [57]. Another study informed that leukopenia might be due to the fact of increased leukocyte mobilization to protect the body in copper-damaged tissue [58]. Moreover, lymphocytopenia could be attributed to the weakening of the immune system caused by Cu or by oxidative stress release due to the fact of Cu poisoning [59,60]. Another experiment stated that excess dietary supplementation levels with nano-Cu led to the reduction of WBCs, Hb, hematocrit, and RBCs in chickens [61]. Dietary inclusion of vitamins C and E, singularly or in combination with Cu, resulted in a correction and improvement in the values of TLC, lymphocytes, heterophils, and monocytes. The associations between metal toxicity and vitamins’ protective effects have been formerly reported [54,55,56]. Both vitamins (i.e., C and E) activate the phagocyte population and immunostimulants or significantly protect WBC against hydrogen peroxide by scavenging [62,63]. 

Concerning the biochemical results in the current inquiry, significant hypoglycemia was recorded in the blood of CuSO_4_-intoxicated birds. This finding was attributed to a diminution in feed intake and agrees with previously obtained results [10,49,56]. Moreover, hypoglycemia indicates a depletion of energy resources (glycogen) and, consequently, deterioration of the organism’s state [64]. Renal function tests in the current work revealed an increase in serum uric acid and creatinine level in the blood of CuSO_4_-intoxicated birds at the sixth week. These elevations indicate a renal impairment (nephrotoxicity) as a result of the alterations in the tubular reabsorption threshold, renal blood flow, and glomerular infiltration rate [65], which suggests that the kidney cannot excrete these products due to the fact of impaired kidney function [66]. Other studies reported that high creatinine level happens due to the fact of severe muscle or renal damage [67], or it indicates signs of renal failure [68]. In birds, uric acid is the main final product of nitrogen catabolism [69]. Raised serum uric acid levels are related to the toxic effects from Cu and kidney failure due to the impact of copper metabolites [53,56,67]. Elevation in the concentration of uric acid in the blood might be confirmed lipid peroxidation caused by Cu toxicity, causing a disturbance in kidney excretion function [12,70]. Another study stated significantly higher uric acid concentrations in the serum of broilers given high dietary copper levels [71]. These findings were confirmed by the histopathological findings in the kidney, which showed extensive necrosis of the renal tubular epithelial, hypercellularity and lymphocytic infiltrations of glomeruli, extensive hemorrhages, hemolysis, and hyaline cast inside some tubular lumina. Some studies conducted in numerous animal models have shown that Cu toxicity causes severe pathological findings in kidneys [46,53,72,73]. In addition, Rasool et al. [74] revealed degenerative and necrotic changes in the kidneys of birds fed CuSO4. Addition of vitamins C and E with copper reduced serum creatinine and uric acid levels compared with the copper-intoxicated group. This might be because of the nephroprotective effect of these vitamins due to the fact of their antioxidant effect. Several investigations have revealed that vitamins C and E act synergistically [30]. This was confirmed pathologically, where the kidney displayed moderate nephritic changes with moderate renal lesions in the CuSO_4_ + vitamin C group, mild degenerative changes with a few regenerative attempts in the CuSO_4_ + vitamin E group, great regenerative attempts in all nephron segments, and the interstitium restored its normal histomorphologic picture in the CuSO_4_ + vitamin C + vitamin E group.

Concerning oxidant and antioxidant biomarkers in the current study, Cu toxicity induced lipid peroxidation, as evidenced by an increased serum MDA concentration, with diminished GSH-Px and SOD activities. Oxidative damage is one of the hallmarks of copper toxicity [75]. Previous studies disclosed that copper toxicity increased MDA production and decreased antioxidant activity in broiler blood [61,76,77]. Increased MDA in copper-treated broilers has been associated with excessive production of ROS or ROS accretion due to the ineffectiveness of the antioxidant system after long-term exposure [78]. The declined SOD and GSH-Px activity could be explained because of the efficiency of antioxidant enzymes in the detoxification of the lipid peroxidation products due to the high concentration of copper in the blood and liver [11]. The fall in the levels of these enzymes will consequently upsurge ROS [79,80]. Supplementation of vitamin C or E alone or in combination with Cu-intoxicated birds revealed enhancement in oxidative stress and antioxidant markers. This was demonstrated by a significantly reduced MDA level with a significant rise in GSH-Px and SOD activities compared to the Cu-intoxicated group. This improvement was more pronounced in broilers given a combination of vitamins (CuSO_4_ + vitamin C + vitamin E group) in comparison to other supplemented groups. However, the protective effect of vitamins for copper-induced oxidative stress was not entirely similar to that of the MDA level, and the antioxidant enzyme activity did not return to the normal control values. Alleviating the effects of both vitamins may increase membranes’ ROS scavenging and their consequent reduction into hydroperoxides, restoring antioxidant enzyme activities [24]. Another recent study reported that lipid peroxidation and hydrogen peroxide production levels were significantly reduced in CuSO_4_-fed fish supplemented with vitamin E [33]. Other investigators have been informed on the ameliorating effects of vitamin C or E in metal-induced toxicity [12,81,82].

## 5. Conclusions

It could be concluded that co-supplementation of vitamins C and E, singularly or in combination, in chickens with CuSO_4_-induced toxicity displayed an enhancement in the hematological and biochemical parameters. Moreover, their supplementation mitigated oxidative stress as well as the histopathological alterations in the kidney. The combination of vitamins C and E showed more beneficial effects due to the fact of their synergistic activity in normalizing the levels of most assessed parameters.

## Figures and Tables

**Table 1:** Proximate and chemical composition of the basal diets (%).

Ingredients	Starter Stage (1–10 Day)	Grower Stage (11–22 Day)	Finisher Stage (23–42 Day)
Soybean meal, 48%	34.65	28.1	24.9
Corn gluten, 60%	1.5	3	3
Yellow corn	58.1	62.1	63.6
Wheat bran	-	1.10	1.80
Soy oil	2.00	2.00	3.26
Calcium carbonate	1.00	1.00	1.00
Calcium dibasic phosphate	1.80	1.70	1.50
Premix *	0.300	0.300	0.300
Common salt	0.300	0.300	0.300
DL-Methionine, 98%	0.180	0.140	0.110
Lysine, Hcl, 78%	0.160	0.160	0.130
Anti-mycotoxin	0.100	0.100	0.100
Proximate composition (%)			
ME, Kcal/Kg	3047.53	3090.13	3178.59
Crude protein	22.14	20.40	19.07
Crude fiber	2.60	2.61	2.63
Fat	4.50	4.61	5.87
Available P	0.49	0.46	0.42
Calcium	0.96	0.93	0.87
Lysine	1.38	1.21	1.09
Methionine	0.56	0.49	0.47

* Premix per kg of diet: vitamin D3, 200 IU; vitamin A, 1500 IU; vitamin E, 10 mg; vitamin K3, 0.5 mg; thiamine, 1.8 mg; riboflavin, 3.6 mg; pantothenic acid, 10 mg; folic acid, 0.55 mg; pyridoxine, 3.5 mg; niacin, 35 mg; cobalamin, 0.01 mg; biotin, 0.15 mg; Cu, 8 mg; Fe, 80 mg; Zn, 40 mg; Mn, 60 mg; Se, 0.15 mg; I, 0.35 mg. ME: metabolizable energy; P: phosphorus.

**Table 2:** Impact of single or combined supplementation of vitamin C and E on the erythrogram of CuSO_4_-intoxicated broiler chickens (mean ± SD, *n* = 5).

	Parameters	CON	CuSO_4_	CuSO_4_ + vitamin C	CuSO_4_ + vitamin E	CuSO_4_ + vitamin C + vitamin E	*p*-Value
At the 3rd week	RBCs (×10^6^/µL)	2.15 ± 0.068 ^a^	1.85 ± 0.094 ^b^	2.08 ± 0.058 ^a^	2.09 ± 0.066 ^a^	2.11 ± 0.052 ^a^	0.00
	Hb (g/dL)	9.73 ± 0.228 ^a^	8.05 ± 0.545 ^c^	9.00 ± 0.158 ^b^	9.15 ± 0.145 ^b^	9.41 ± 0.303 ^a,b^	0.00
	HCT (%)	25.1 ± 0.47 ^a^	21.5 ± 1.32 ^b^	23.9 ± 0.54 ^a^	24.1 ± 0.66 ^a^	24.5 ± 0.61 ^a^	0.00
	MCV (fL)	116 ± 1.8	116 ± 2.5	115 ± 1.8	1151 ± 1.4	116 ± 1.0	0.59
	MCH (Pg)	45.1 ± 1.83	43.4 ± 1.77	43.1 ± 1.06	43.8 ± 1.13	44.5 ± 0.48	0.12
	MCHC (%)	38.8 ± 1.10	37.3 ± 0.97	37.6 ± 0.71	38.0 ± 0.82	38.3 ± 0.73	0.05
At the 6th week	RBCs (×10^6^/µL)	2.79 ± 0.124 ^a^	2.42 ± 0.045 ^d^	2.55 ± 0.049 ^c,d^	2.60 ± 0.088 ^b,c^	2.70 ± 0.077 ^a,b^	0.00
	Hb (g/dL)	10.98 ± 0.184 ^a^	9.54 ± 0.383 ^c^	9.93 ± 0.109 ^b,c^	10.18 ± 0.268 ^b^	10.68 ± 0.226 ^a^	0.00
	HCT (%)	28.4 ± 0.56 ^a^	26.0 ± 0.73 ^c^	27.1 ± 0.51 ^b^	27.5 ± 0.60 ^a,b^	28.1 ± 0.84 ^a,b^	0.00
	MCV (fL)	101 ± 1.6	107 ± 1.6	106 ± 1.7	106 ± 1.7	103 ± 1.0	0.51
	MCH (Pg)	40.1 ± 2.55	38.7 ± 1.11	38.6 ± 0.84	39.1 ± 0.89	39.5 ± 0.94	0.37
	MCHC (%)	38.2 ± 0.51 ^a^	36.1 ± 0.69 ^c^	36.5 ± 0.53 ^c^	37.0 ± 0.51 ^b,c^	37.9 ± 1.04 ^a,b^	0.00

^a,b,c,d^ Means carrying different superscripts are significantly different at *p* < 0.05. RBCs: Red blood cells, Hb: hemoglobin, HCT: hematocrit, MCV: mean corpuscular volume, MCH: mean corpuscular hemoglobin, MCHC: mean corpuscular hemoglobin concentration.

**Table 3:** Effect of single or combined supplementation of vitamin C and E on the leukogram of CuSO_4_-intoxicated broiler chickens (mean ± SD, *n* = 5).

	Parameters	CON	CuSO_4_	CuSO_4_ + Vitamin C	CuSO_4_ + Vitamin E	CuSO_4_ + Vitamin C + Vitamin E	*p*-Value
At the 3rd week	WBCs (×10^3^/µ L)	20.4 ± 1.33 ^a^	17.2 ± 1.21 ^c^	18.0 ± 0.88 ^b,c^	18.7 ± 0.77 ^a,b,c^	19.7 ± 1.33 ^a,b^	0.001
	Heterophils (×10^3^/µ L)	6.79 ± 0.459	6.23 ± 0.393	6.30 ± 0.312	6.56 ± 0.379	6.70 ± 0.459	0.09
	Lymphocytes (×10^3^/µ L)	10.54 ± 0.425 ^a^	8.32 ± 0.927 ^c^	9.03 ± 0.654 ^b,c^	9.41 ± 0.685 ^a,b,c^	0.15 ± 0.758 ^a,b^	0.00
	Monocytes (×10^3^/µ L)	1.59 ± 0.157 ^a^	1.16 ± 0.340 ^b^	1.27 ± 0.071 ^a,b^	1.30 ± 0.084 ^a,b^	1.44 ± 0.105 ^a,b^	0.007
	Eosinophils (×10^3^/µ L)	0.980 ± 0.0604	1.03 ± 0.288	0.970 ± 0.0484	0.982 ± 0.1042	0.992 ± 0.1308	0.95
	Basophils (×10^3^/µ L)	0.506 ± 0.0493	0.468 ± 0.0497	0.472 ± 0.0396	0.472 ± 0.0438	0.486 ± 0.0461	0.57
At the 6th week	WBCs (×10^3^/µ L)	21.1 ± 1.90 ^a^	15.8 ± 1.68 ^c^	16.8 ± 0.87 ^c^	17.9 ± 0.58 ^b,c^	19.9 ± 1.25 ^a,b^	0.00
	Heterophils (×10^3^/µ L)	7.12 ± 0.674 ^a^	5.26 ± 1.088 ^b^	5.99 ± 0.321 ^a,b^	6.14 ± 0.571 ^a,b^	6.75 ± 0.447 ^a^	0.001
	Lymphocytes (×10^3^/µ L)	10.84 ± 0.993 ^a^	7.86 ± 0.616 ^b^	8.16 ± 0.532 ^b^	9.01 ± 0.564 ^b^	10.20 ± 0.700 ^a^	0.00
	Monocytes (×10^3^/µ L)	1.60 ± 0.150 ^a^	1.18 ± 0.340 ^b^	1.29 ± 0.071 ^a,b^	1.34 ± 0.084 ^a,b^	1.45 ± 0.102 ^a,b^	0.008
	Eosinophils (×10^3^/µ L)	1.00 ± 0.090	1.05 ± 0.388	0.980 ± 0.0484	0.992 ± 0.1042	1.08 ± 0.130	0.83
	Basophils (×10^3^/µ L)	0.518 ± 0.0476 ^b^	0.450 ± 0.0509	0.462 ± 0.0396	0.472 ± 0.0438	0.506 ± 0.0461	0.07

^a,b,c^ Means carrying different superscripts are significantly different at *p* < 0.05.

**Table 4:** Effect of single or combined supplementation of vitamin C and E on the serum glucose and renal biomarkers of CuSO4-intoxicated broiler chickens (Mean ± SD, *n* = 5).

	Parameters	CON	CuSO_4_	CuSO_4_ + Vitamin C	CuSO_4_ + Vitamin E	CuSO_4_ + Vitamin C + Vitamin E	*p* Value
At the 3rd week	Glucose (mg/dL)	157 ± 10.01 ^a^	117 ± 7.20 ^b^	127 ± 8.00 ^a,b^	136 ± 7.5 ^a,b^	150 ± 9.7 ^a^	0.009
	Uric acid (mg/dL)	3.29 ± 0.553	3.70 ± 0.266	3.19 ± 0.158	3.39 ± 0.503	3.14 ± 0.479	0.25
	Creatinine (mg/dL)	0.508 ± 0.0679	0.618 ± 0.1160	0.566 ± 0.0585	0.532 ± 0.0438	0.528 ± 0.0759	0.13
At the 6th week	Glucose (mg/dL)	159 ± 17.9 ^a^	122 ± 9.8 ^b^	135 ± 13.6 ^a,b^	143 ± 11.3 ^a,b^	152 ± 14.0 ^a^	0.003
	Uric acid (mg/dL)	4.41 ± 0.570 ^b^	5.18 ± 0.362 ^a^	4.85 ± 0.352 ^a,b^	4.83 ± 0.651 ^a,b^	4.54 ± 0.405 ^b^	0.008
	Creatinine (mg/dL)	0.614 ± 0.0665 ^b^	0.784 ± 0.0971^a^	0.688 ± 0.0664 ^a,b^	0.624 ± 0.0646 ^b^	0.586 ± 0.1059 ^b^	0.009

^a,b^ Means carrying different superscripts are significantly different at *p* < 0.05.

**Table 5:** Impact of single or combined supplementation of vitamin C and E on the serum oxidative stress indicator and antioxidant status of CuSO4-intoxicated broiler chickens (mean ± SD, *n* = 5).

	Parameters	CON	CuSO_4_	CuSO_4_ + Vitamin C	CuSO_4_ + Vitamin E	CuSO_4_ + Vitamin C + Vitamin E	*p*-Value
At the 3rd week	MDA (nmol/mL)	1.28 ± 0.177 ^c^	1.78 ± 0.094 ^a^	1.64 ± 0.134 ^a,b^	1.53 ± 0.182 ^a,b,c^	1.44 ± 0.131 ^b,c^	0.00
	GSH-Px (U/mL)	37.3 ± 5.71	30.3 ± 3.50	31.5 ± 3.13	34.0 ± 8.00	36.1 ± 6.38	0.07
	SOD (U/mL)	4.04 ± 0.114 ^a^	2.48 ± 0.211 ^c^	2.95 ± 0.124 ^b^	3.08 ± 0.174 ^b^	3.10 ± 0.234 ^b^	0.00
At the 6th week	MDA (nmol/mL)	1.34 ± 0.157 ^c^	2.01 ± 0.222 ^a^	1.84 ± 0.152 ^a,b^	1.74 ± 0.147 ^a,b^	1.65 ± 0.249 ^b^	0.00
	GSH-Px (U/mL)	57.0 ± 6.70 ^a^	37.6 ± 3.60 ^c^	43.6 ± 3.57 ^b^	46.3 ± 11.23 ^b^	50.4 ± 4.97 ^a,b^	0.00
	SOD (U/mL)	4.99 ± 0.234 ^a^	2.91 ± 0.177 ^d^	3.24 ± 0.350 ^c,d^	3.61 ± 0.165 ^b,c^	3.85 ± 0.350 ^b^	0.00

^a,b,c,d^ Means carrying different superscripts are significantly different at *p* < 0.05. MDA: Malondialdehyde, SOD: superoxide dismutase, GSH-Px: glutathione peroxidase.

## Data Availability

Data sharing not applicable.
